# Interconnected Micro, Meso, and Macro Porous Activated Carbon from Bacterial Nanocellulose for Superior Adsorption Properties and Effective Catalytic Performance

**DOI:** 10.3390/molecules25184063

**Published:** 2020-09-05

**Authors:** Arnon Khamkeaw, Tatdanai Asavamongkolkul, Tianpichet Perngyai, Bunjerd Jongsomjit, Muenduen Phisalaphong

**Affiliations:** 1 Bio-Circular-Green-economy Technology & Engineering Center, BCGeTEC, Department of Chemical Engineering, Faculty of Engineering, Chulalongkorn University, Bangkok 10330, Thailand; arnon.k1231@gmail.com (A.K.); tatdanai.asa@gmail.com (T.A.); tianpichet.p@hotmail.com (T.P.); bunjerd.j@chula.ac.th (B.J.); 2 Center of Excellence on Catalyst and Catalytic Reaction Engineering, Department of Chemical Engineering, Faculty of Engineering, Chulalongkorn University, Bangkok 10330, Thailand

**Keywords:** activated carbon, bacterial cellulose, catalyst, ethanol dehydration, adsorption

## Abstract

The porous carbon (bacterial cellulose (BC)-activated carbon (AC)(BA)) prepared via two-step activation of bacterial nanocellulose by treatments with potassium hydroxide (KOH) and then phosphoric acid (H_3_PO_4_) solutions showed superior adsorption properties and effective performance as catalyst support. BC-AC(BA) had an open and interconnected multi-porous structure, consisting of micropores (0.23 cm^3^/g), mesopores (0.26 cm^3^/g), and macropores (4.40 cm^3^/g). The BET surface area and porosity were 833 m^2^/g and 91.2%, respectively. The methylene blue adsorption test demonstrated that BC-AC(BA) was superior in its mass transfer rate and adsorption capacities. Moreover, BC-AC(BA) modified by H_3_PO_4_ treatment showed a significant enhancement of catalytic performance for dehydration of ethanol. At the reaction temperature of 250–400 °C, 30P/BC-AC(BA) gave ethanol conversion at 88.4–100%, with ethylene selectivity of 82.6–100%, whereas, high selectivity for diethyl ether (DEE) at 75.2%, at ethanol conversion of 60.1%, was obtained at the reaction temperature of 200 °C.

## 1. Introduction

Activated carbon (AC) is a carbonaceous material with high porosity, high surface area, high physical and chemical stability, high adsorptive activity, high mechanical strength, high thermal stability, and low cost [1]. These properties lead to a wide variety of industrial applications, such as an adsorbent in an adsorption process [2], catalyst or catalyst support in chemical reactions [3], material for removal of heavy metals from wastewater [4], and separator material in gas separation processes [5]. The production of AC has been prepared from many kinds of organic substances, such as agricultural by-products and residual wastes. Previously, our research works have presented that effective ACs can be prepared from bacterial cellulose (BC) [6,7]. BC has emerged as a renewable material in recent years. BC is a cellulosic biomaterial with the formula of (C_6_H_10_O_5_)_n_. It is produced by *Acetobacter xylinum* bacteria using sugars and other carbohydrate substrates as carbon sources. BC has a nanoporous structure formed by micro- and nanocellulose fibers with unique properties, including its high porosity, high degree of crystallinity, high surface area for adsorption, high tensile strength, high water-retention capacity, low solubility, and resistance to organic solvents [8]. BC is of a high purity of cellulose because its structure does not contain lignin, hemicelluloses, or other complex carbohydrates. Because of these properties, BC should have great potential to be used as source material for highly efficient activated carbon. During the carbonization process, high amounts of oxygen-containing functional groups in cellulose tend to be eliminated as H_2_O, CO_2_, and CO, resulting in BC-derived activated carbon with high porosity and high surface area [6,7]. BC-derived activated carbon (BC-AC) was used as an adsorbent for removing methylene blue (MB) from aqueous solutions [6] and was applied as green catalyst support for ethanol conversion to ethylene in ethanol dehydration reaction [7]. BC-AC showed high adsorption properties and effective performance as catalyst support. However, its structure with very small pore sizes could limit the mass transfer efficiency. The diffusion in small pores often results in a limitation of mass transfer, which could make the molecules of reactants and products difficult to diffuse in and out of the catalysts. In addition, the micro/mesoporous structure of BC-AC catalyst could lead to pore blockage by coke deposition, resulting in catalyst deactivation [9]. To overcome this mass transfer limitation, this study aimed to develop BC-AC with an improved porous structure, high porosity and high surface area.

From our previous works, BC-AC was synthesized by one-step chemical activation, using either potassium hydroxide (KOH) or phosphoric acid (H_3_PO_4_). By using KOH as an activating agent [10], the BC-AC showed high porosity with meso/macroporous structure, but it had low surface area. By using phosphoric acid (H_3_PO_4_) as an activating agent [6,7], the BC-AC exhibited a high surface area with micro/meso porous structure. The synthesis of highly porous AC from BC with an interconnected micro-, meso-, and macroporous structure was firstly introduced in this study, in which the two step chemical activations using both H_3_PO_4_ and KOH as activating agents were performed to improve the porous structure. The use of H_3_PO_4_ is intended to provide mesopores with high surface area, whereas the use of KOH aims to provide high porosity with large pore width. Therefore, by applying this two-step chemical activation, an interconnected micro-, meso-, and macroporous AC derived from BC with high absorption rate combined with high absorption capacity could be developed. The adsorptive and mass transfer performances of the altered ACs were investigated by MB adsorption. Then, ACs were modified by H_3_PO_4_ loading for improving surface acidity and further applied as a solid acid catalyst for the ethanol dehydration reaction.

## 2. Results and Discussion

### 2.1. Characterizations

#### 2.1.1. SEM

ACs derived from BCs (BC-AC) were prepared by one-step and two-step chemical activation with H_3_PO_4_ (A) and/or KOH (B) as activating agent using an incipient wetness technique. The samples obtained from one-step activation are denoted as BC-AC(X), where X is the activating agent, i.e., BC-AC(A) refers to an AC derived from BC with H_3_PO_4_ (A) activation. The samples obtained from two-step activation are denoted as BC-AC(YZ), where Y is the first activating agent in first step activation, and Z is the second activating agent in second step activation, i.e., BC-AC(AB) refers to an AC derived from BC by using H_3_PO_4_ (A) as first activating agent in first step activation and KOH (B) as second activating agent in second step activation.

**Figure 1:**
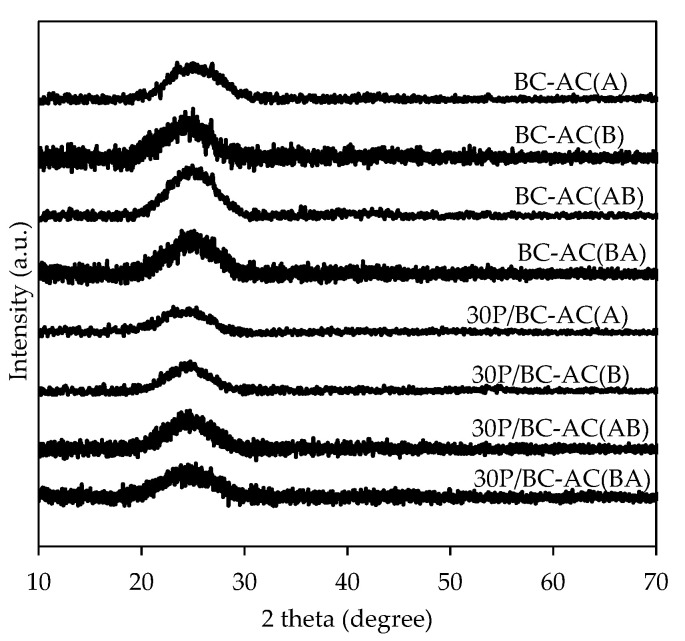
XRD patterns of bacterial cellulose-activated carbons (BC-ACs) and 30P/BC-ACs.

**Figure 2:**
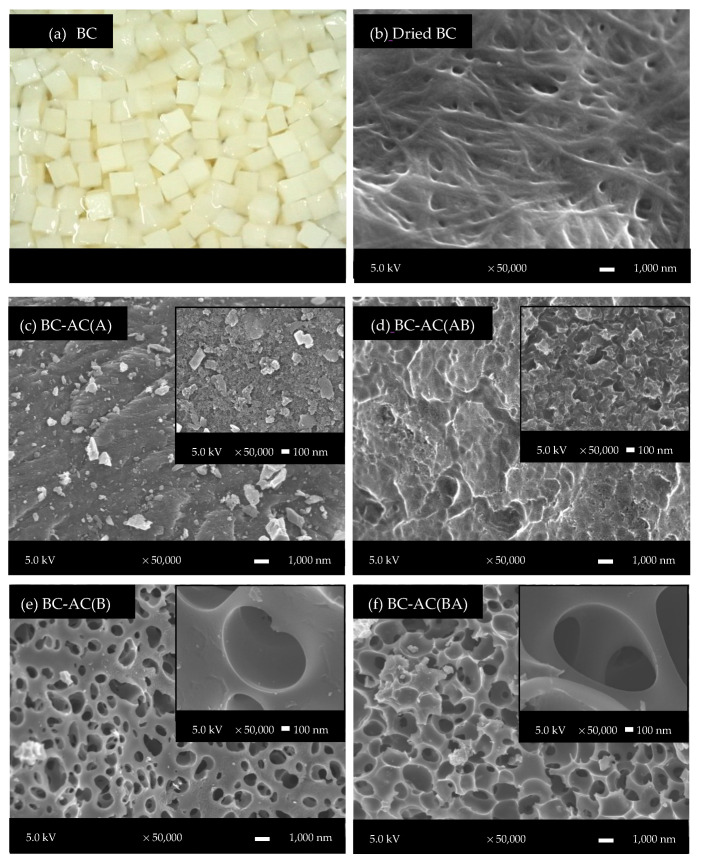
Images of (**a**) BC and SEM micrograph of surface morphology of (**b**) dried BC and (**c**–**f**) BC-ACs.

According to the XRD patterns of BC-ACs, all samples displayed only a diffraction peak at around 25°, indicating that all samples are amorphous materials composed of aromatic carbon sheets (Figure 1). Figure 2 shows the surface morphology and porous structure of BC-ACs. Before activating, the BC hydrogel has ultra-fine network structure consists of nano- and microfibers [11]. This structure is preserved even after being dried. After activating with strong acid and strong base at high temperature, the surface morphology of the BC has changed because the micropores, mesopores, and macropores were created in the structure resulting in the higher surface area. However, the raw form of BC that has nano- and microfibers network structure and high porosity is also inherited to the BC-ACs resulting in the high porosity. Under one-step chemical activation, the obtained BC-AC(A) from the activation of BC with H_3_PO_4_ shows rough surfaces with mainly micropores and mesopores, whereas the obtained BC-AC(B) from the activation of BC with KOH was mainly composed of meso/macropores with large and deep cavities. Therefore, H_3_PO_4_ and KOH agents played different roles on the structure development of the BC-ACs. The using H_3_PO_4_ as an activating agent could lead to bond cleavage and formation of cross-linked structure. When the concentrated H_3_PO_4_ reacted with cellulose, the cellulose is depolymerized and linked to H_3_PO_4_ under the form of cellulose phosphate esters [12]. During the activation process, the oligomerized H_3_PO_4_ that become part of the carbon matrix transformed into P_2_O_5_ and water. The sublimation of P_2_O_5_ leads to the generation of pores; meanwhile, the evaporation of water molecules leads to the development of porosity [13]. On the other hand, KOH activation involves mainly fragmentation and solubilization of raw structure of precursor via a reduction reaction while leaving potassium-containing atoms within the carbon network. The potassium compound products can diffuse and intercalate into the carbon lamellae to induce the separation of layer and pore development [14]. This increases the net volume of the precursor and lowers its density. This may be associated with larger porous structure development that results in higher porosity. Under two-step chemical activation, BC-AC(AB), which was obtained from BC-AC(A) secondly chemical activated with KOH, presented more macropores, whereas the numbers of micropores and mesopores decreased due to the expansion of some micro/mesopores into macropores. The collapse of pore structure of BC-AC(AB) after the carbonization process at 500 °C was observed. On the other hand, BC-AC(BA) obtained from BC-AC(B) secondly chemical activated with H_3_PO_4_ presented micropores and mesopores restricted to surface of macropores. Therefore, a well-defined open and interconnected micro-, meso-, and macroporous structure can be created by two-step activation by KOH solution and followed by H_3_PO_4_ solution.

#### 2.1.2. Pore Structure and Surface Area Analysis

The N_2_ physisorption was used to determine micropore and mesopore characteristics (pore size diameter 0–50 nm). Meanwhile, the mercury porosimetry was used to determine macropore characteristics (pore size diameter 50–500,000 nm). Table 1 shows the surface area and pore volume of BC-ACs. BC-AC(A) exhibited high BET surface area of 1316 m^2^/g, which was significantly higher than BC-AC(B). The pore-structure of BC-AC(A) has a porosity of 65.0%, consisted of micropores at volume of 0.17 cm^3^/g and mesopores at volume of 0.71 cm^3^/g. BC-AC(A) exhibited lower macropore volume and less porosity than BC-AC(B). BC-AC(B) has a higher porosity of 79.7% and consisted of high macropores at volume of 3.3 cm^3^/g. Owing to the effect of activating agents, H_3_PO_4_ produced AC with high surface area of micropores and mesopores [6], whereas KOH produced AC with macroporous structure and high porosity [10]. After the second chemical activation with KOH, the BET surface area and the micropore and mesopore volume of BC-AC(AB) decreased, whereas the macropore volume and porosity of BC-AC(AB) increased. Therefore, the macropores could be generated in-situ after the activation of BC-AC(A) with KOH. The results also demonstrate that, after the second activation of BC-AC(B) with H_3_PO_4_, the BET surface area, micropore and mesopore volume, macropore volume, and porosity of BC-AC(BA) significantly increased as compared to BC-AC(B) due to the enlarged macropores and the formation of micro/mesopores restricted to the surface of macropores during the carbonization process. The high macropore volume of 4.4 cm^3^/g and porosity of 91.2% were obtained.

**Figure 3:**
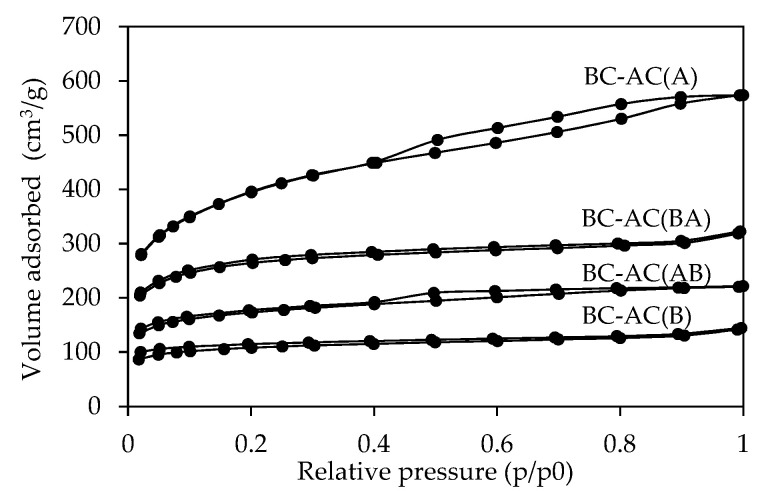
The N_2_ adsorption-desorption isotherms of BC-ACs.

N_2_ adsorption-desorption isotherms are shown in Figure 3. The BC-AC(A) exhibited a complete hysteresis loop at the relative pressure ≥0.4, whereas the BC-AC(AB) lacked closure of the hysteresis loop below a relative pressure of 0.4, which might be due to the expansion of a mesoporous to macroporous structure [15]. No hysteresis loops between the adsorption/desorption isotherms exist in BC-AC(B) and BC-AC(BA) due to the very high proportion of macropore volume in the porous structure [16]. According to International Union of Pure and Applied Chemistry (IUPAC) classification of physisorption isotherms [17], the isotherm of BC-AC(A) was type IV, which indicates a mesoporous structure, whereas the isotherms of the BC-AC(AB), BC-AC(B), and BC-AC(BA) were type II, which are associated with a macroporous structure.

**Figure 4:**
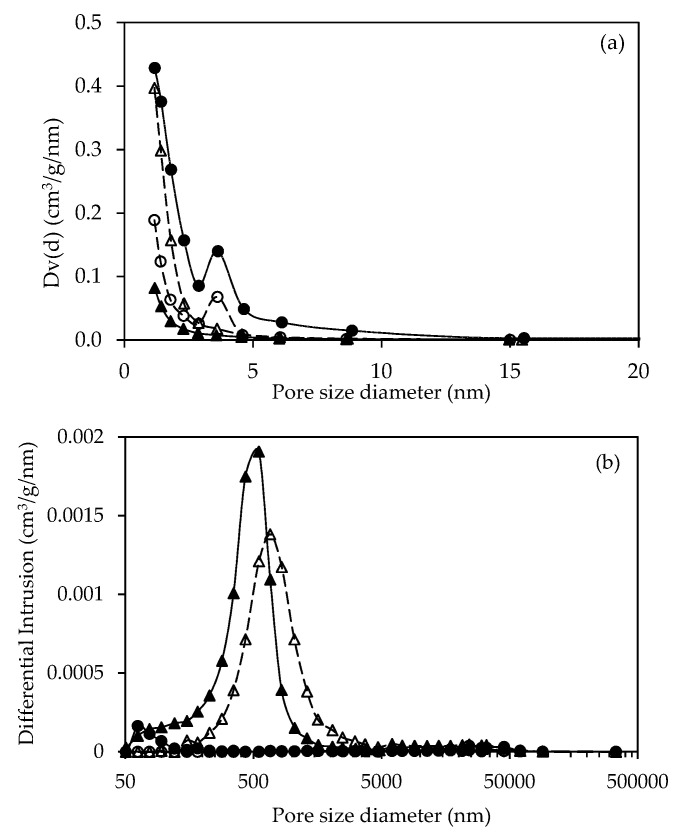
The pore size distribution of BC-AC(A) (•), BC-AC(B) (▲), BC-AC(AB) (○), and BC-AC(BA) (Δ) from N_2_ physisorption (**a**) and mercury porosimetry (**b**).

Figure 4 shows the pore size distribution of BC-ACs determined using N_2_ physisorption and mercury porosimetry. BC-ACs show a pore size distribution range of micropores and mesopores at 1.2–15.5 nm (Figure 4a). BC-AC(A) has the highest surface area, followed by BC-AC(BA), BC-AC(AB), and BC-AC(B), respectively. The macropores were verified by the mercury porosimetry method, as shown in Figure 4b. BC-AC(B) and BC-AC(BA) had much larger macropore volumes than those of BC–-AC(A) and BC-AC(AB). The result demonstrates that the two-step chemical activations can promote multiporous structures with high porosity. The surface area and porosity of BC-ACs were compared with AC derived from jackfruit leaf [18]. Jackfruit leaf is one of natural cellulose fiber sources (without nano structure). At the same activation temperature and activating agent, BC-ACs had significantly higher surface area and porosity. ACs derived from jackfruit leaf had a surface area of 67 and 44 m^2^/g by the activation with H_3_PO_4_ and KOH, respectively, with a porosity of 51 and 36%, respectively, which was relatively low as compared to ACs derived from BC (Table 1). This indicated that the raw nanostructure of BC can promote the high porosity in the porous structure of AC during the activation process.

#### 2.1.3. Acidity

The surface acidity of BC-ACs was modified with H_3_PO_4_ treatment using the incipient wetness technique in order to further apply as solid acid catalysts in an ethanol dehydration reaction. According to a previous study [7], the optimal H_3_PO_4_ loading on BC-AC is 30 wt %. The solid catalysts of 30P/BC-ACs are BC-ACs loaded with 30 wt % H_3_PO_4_ (i.e., 30P/BC-AC(A) refers to BC-AC(A) loaded with 30 wt % of H_3_PO_4_).

The temperature-programmed desorption of ammonia (NH_3_-TPD) was used to determined surface acidity and strength of acid sites of samples. The desorption temperature indicates the acid strength of the sites, with weaker sites desorbing at low temperatures (<250 °C) defined as weak acid sites, while those desorbing at high temperatures (250–500 °C) were defined as moderate to the strong acid sites [19]. Table 2 shows the summary of the number of the acid sites before and after modification of surface acidity with H_3_PO_4_ treatment. Before surface modification of BC-ACs, the amounts of weak acid sites and moderate to strong acid sites depend on the surface area of catalysts, listed in order from high to low, were as follows: BC-AC(A) > BC-AC(BA) > BC-AC(AB) > BC-AC(B). High surface area provides high amount of phosphate groups on the surface, which are formed on the carbon surface during the activation process of BC-ACs. The P-OH groups and phosphate ester and/or phenol groups correspond to weak acid sites, and the H-phosphate groups correspond to moderate to strong characters [20]. After the modification of BC-ACs surface acidity with 30% loading of H_3_PO_4_, the 30P/BC-ACs show higher numbers of weak acid sites and moderate to strong acid sites than BC-ACs. In a comparison between the amount weak acid site and moderate to strong acid sites, the BC-ACs and 30P/BC-ACs had higher amounts of weak acid sites than moderate to strong acid sites. Moreover, the H_3_PO_4_ modification enhanced the ratio of weak/moderate to strong from 1.9–2.0 to 2.8–3.9. It has been reported previously that the catalysts associated with a larger number of weak acid sites have good performance in ethanol dehydration because the ethanol conversion to ethylene occurs on weak acid sites, whereas the oligomerization and alcohol transformation to higher hydrocarbons correspond to strong acid strength [21]. After loading of H_3_PO_4_, the amounts of weak acid sites, listed in order from high to low, were as follows: 30P/BC-AC(A) > 30P/BC-AC(BA) > 30P/BC-AC(AB) > 30P/BC-AC(B). 

#### 2.1.4. FTIR

**Figure 5:**
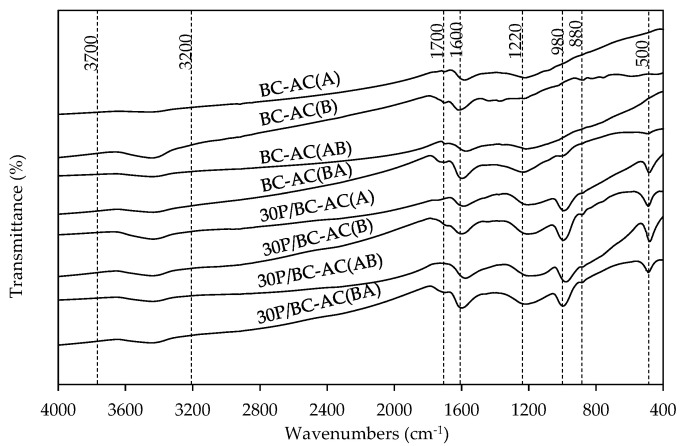
FT-IR patterns of BC-ACs and 30P/BC-ACs.

Figure 5 shows FTIR spectra of BC-ACs and 30P/BC-ACs. The broad band in the region of 3700–3200 cm^−1^ is attributed to O-H stretching vibration of hydroxyl group [6]. The band at approximately ~1700 cm^−1^ is attributed to C=O stretching vibrations of ketones, aldehydes, lactones, or carboxyl groups [22]. The strong band at 1600 cm^−1^ is ascribed to the C–C stretching vibrations from aromatic rings. The broad band at approximately ~1220 cm^−1^ may correspond to C–O stretching in acids, alcohols, phenols, ethers, and/or esters groups [22]. The aromatic character of the carbon is confirmed by the peaks at 880 cm^−1^ associated with aromatic C–H out-of-plane vibrations for different substituted benzene rings, which are evidence of the carbon [22]. The two bands at 980 and 500 cm^−1^ are assigned to the stretching vibrational mode of hydrogen-bonded P=O, to O–C stretching vibrational in P–O–C (aromatic) linkage, and to O=P–OH groups [23]. The presence of P-O and P-OH on the carbon surface can act as Brønsted acid sites, and they play an important role in the acidic character of the carbon catalysts prepared for ethanol dehydration [7]. After the modification of BC-ACs surface with H_3_PO_4_ treatment, the 30P/BC-ACs show higher phosphate group characteristics on the surface at two bands of 980 and 500 cm^−1^, which is consistent with the surface acidity results. 

#### 2.1.5. XPS

**Figure 6:**
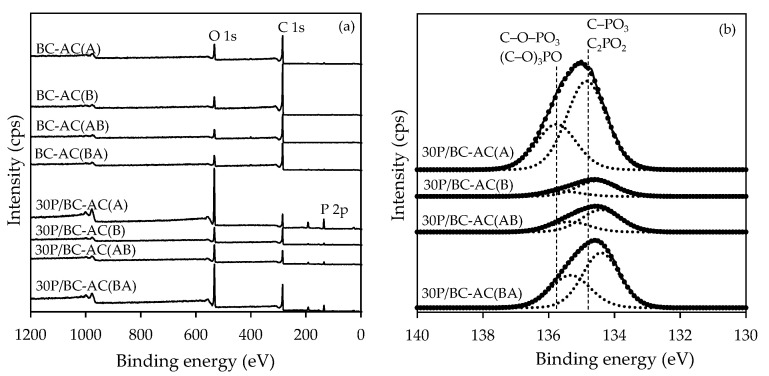
XPS wide scan spectra of all samples (**a**) and deconvolution of XPS spectra P 2p zone of 30P/BC-ACs (**b**).

X-ray photoelectron spectroscopy (XPS) analyses were carried out in order to evaluate the surface element distribution and surface chemical structure of the samples. Figure 6a shows the wide range-scanning spectrum of BC-ACs and 30P/BC-ACs. The spectra of BC-ACs shows clearly C and O peaks, representing the major constituents of surface, while the spectra of 30P/BC-ACs shows major peaks of C, O and small peak of P. These results correspond to the results obtained from FTIR spectroscopy. After modification surface with H_3_PO_4_, the 30P/BC-ACs show P group characteristics on the surface. Mass surface concentrations of BC-ACs and 30P/BC-ACs are shown in Table 3. The 30P/BC-ACs contained considerably higher amounts of P and O as compared to the BC-ACs. The amounts of P on 30P/BC-ACs surface were as follows: 30P/BC-AC(A) > 30P/BC-AC(BA) > 30P/BC-AC(AB) > 30P/BC-AC(B), which corresponded to the quantities of acid sites of 30P/BC-ACs as shown in Table 2. The small amount of Si was detected despite being a non-common constituent of AC derived from cellulose, which could be an inorganic impurity, sometimes detected from the energy dispersive spectrum of AC [24]. In order to describe the P groups bonded to a carbon site after the modification surface with H_3_PO_4_, the XPS spectra of P 2p zone of 30P/BC-ACs was examined. The deconvolution of XPS spectra and the relative amount of phosphorus surface groups are shown in Figure 6b, and Table 4, respectively. The peak at around 135.7 eV is ascribed to P groups bonded to one C atom and three O atoms as in C–O–PO_3_ and (C–O)_3_ PO [20]. The peak at around 134.8 eV is characteristic of C–P bonding as in C–PO_3_ and C_2_PO_2_ groups [20]. The peak position slightly shifted from the maximum of the P 2p band to lower binding energy for 30P/BC-AC(B), 30P/BC-AC(AB), and 30P/BC-AC(BA) with an increase in the proportion of C–PO_3_ and C_2_PO_2_ groups [25]. These acidic compounds were produced from the H_3_PO_4_ treatment. It was suggested that phosphates strongly bound to carbon lattice via C–O–P bonding; therefore, they are chemically and thermally stable [26]. This was again confirmed that the modification of surface acidity by the H_3_PO_4_ treatment effectively created Brønsted acid sites on carbon surface to be used as an effective acid catalyst for dehydration of ethanol.

### 2.2. Mass Transfer and Adsorption Capacity

**Figure 7:**
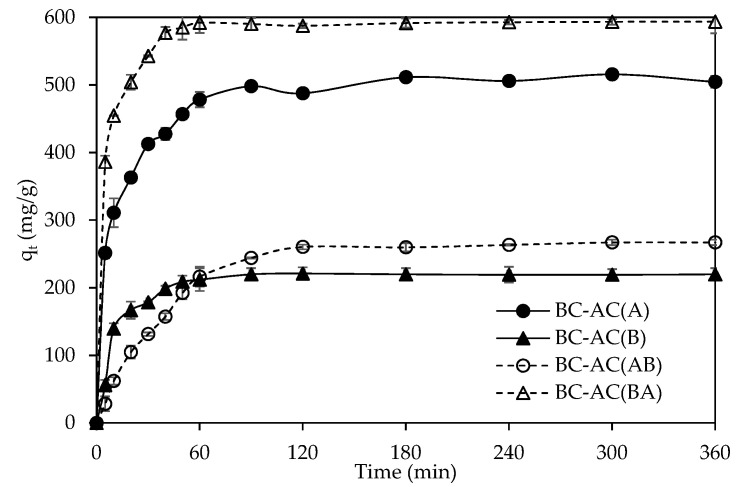
The adsorption kinetics of BC-AC(A) (●), BC-AC(B) (▲), BC-AC(AB) (O), and BC-AC(BA) (Δ) from methylene blue (MB) adsorption at initial concentration of 600 mg/L.

To understand the diffusion in the porous structure of BC-ACs, the mass transfer performance of BC-ACs was studied in the adsorption of MB at the high initial MB concentration of 600 mg/L. Figure 7 shows the experimental kinetic data of all samples. A rapid initial rate of adsorption during 0–60 min was observed, which is likely due to the high MB concentration gradient between solution and adsorbents, creating fast transfer of solute ions to the surface of the adsorbents, and also the availability of the specific surface area of adsorbent sites [27]. BC-AC(BA) shows the best mass transfer performance with fastest adsorption rate in the adsorption of MB compared with BC-AC(A), BC-AC(AB), and BC-AC(B). Because BC-AC(BA) had the highest porosity with an open pore network structure, consisting of micro-, meso-, and macropores, it facilitated the diffusion of MB molecules in porous structure [28], resulting in the fastest mass transfer rate. The molecules of MB could more easily diffuse into the porous structure of BC-AC(BA). Adsorption equilibrium was reached after approximately 60 min for BC-AC(BA), ≈90 min for BC-AC(B), ≈120 min for BC-AC(AB), and ≈180 min for BC-AC(A). The faster adsorption rate was due to the higher macropore volume and higher porosity, which promoted the mass transfer of MB from the bulk solution into the interior of the adsorbents. Moreover, BC-AC(BA) had the highest adsorption capacity, with a maximum value of 593.4 mg/g. Although the BC-AC(A) had a larger surface area than BC-AC(BA), the structure with smaller pore sizes and lower porosity could limit the mass transfer efficiency, especially for the diffusion into the interior of BC-AC(A), leading to less adsorption capacity as compared to BC-AC(BA). 

### 2.3. Catalytic Ethanol Dehydration Performance 

**Figure 8:**
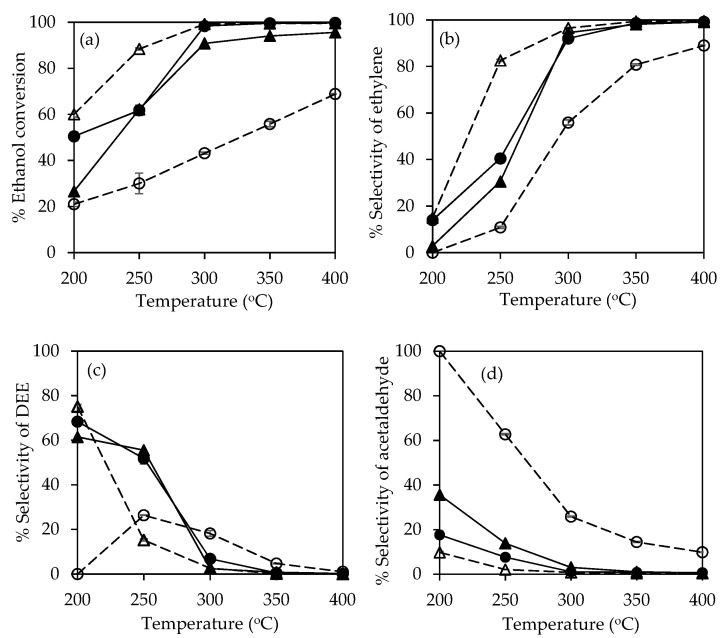
Ethanol conversion (**a**), selectivity of ethylene (**b**), selectivity of diethyl ether (DEE) (**c**), and selectivity of acetaldehyde (**d**) in ethanol dehydration at reaction temperatures of 200–400 °C using the BC-AC(A) (●), BC-AC(B) (▲), BC-AC(AB) (O), and BC-AC(BA) (Δ) catalysts.

Figure 8a presents the ethanol conversion of 30P/BC-AC catalysts. Similar effects related to reaction temperature and acidity of catalyst on the ethanol conversion have been previously reported [29]. The ethanol conversion of all 30P/BC-AC catalysts increased with increased reaction temperature from 200 °C to 400 °C because the ethanol dehydration reaction is an endothermic reaction that favors high temperature condition for the conversion of ethanol to ethylene [7]. The high conversion of ethanol at 100% was obtained by using 30P/BC-AC(BA) and 30P/BC-AC(A) at a reaction temperature of 350–400 °C. At lower reaction temperatures (below 350 °C), 30P/BC-AC(BA) shows a higher conversion of ethanol than the other catalysts. Overall, 30P/BC-AC(BA) gave the highest catalytic activity, i.e., 60.1% of ethanol conversion at reaction temperature of 200 °C, and 88.4–100% of ethanol conversion at reaction temperature of 250–400 °C. Although 30P/BC-AC(A) had more weak acid sites as compared to 30P/BC-BC500(BA), it was shown that, with the higher macropore volume and porosity, the enhanced mass transfer inside the catalyst could be promoted, resulting in effective overall catalytic activities of 30P/BC-BC500(BA). Owing to suitable porous structure, the reactants can easily diffuse into the catalyst pores and are converted to products at active sites [30], and the obtained products can easily diffuse out of the catalyst. The interconnected micro-, meso-, and macroporous structure gives the catalyst with a high number of active sites and high internal diffusion. 

All 30P/BC-AC catalysts show the highest ethylene selectivity at the reaction temperature of 400 °C (Figure 8b), at which the ethylene selectivity of 100%, 100%, 99.1%, and 89.0% were obtained from BC-AC(BA), BC-AC(A), BC-AC(B), and BC-AC(AB), respectively. However, at lower reaction temperatures below 400 °C, the ethylene yield obtained with 30P/BC-AC(BA) was considerably higher than those obtained by using 30P/BC-AC(A), 30P/BC-AC(B), and 30P/BC-AC(AB). Overall, the order of catalytic performance from high to low was as follows: 30P/BC-AC(BA) > 30P/BC-AC(A) > 30P/BC-AC(B) > 30P/BC-AC(AB). The 30P/BC-AC(BA) gave the selectivity of ethylene of 82.6–99.4% at the reaction temperature range of 250–350 °C. The selectivity of ethylene of all 30P/BC-AC catalysts decreased with decreased reaction temperature due to the more conversion of ethanol into DEE and acetaldehyde. At 200 °C, the selectivity for DEE (Figure 8c) was as follows: 61.5% for 30P/BC-AC(B), 68.4% for 30P/BC-AC(A), and 75.2% for 30P/BC-AC(BA), whereas the selectivity of acetaldehyde as the dominant product from 30P/BC-AC(AB) was 100% at 200 °C (Figure 8d). However, because of the low ethanol conversion of 30P/BC-AC(AB) at 200 °C, the acetaldehyde yields were quite low (21.0%).

Table 5 shows the yield of ethylene, diethyl ether (DEE), and acetaldehyde (MeCHO) from ethanol dehydration at 200, 250, 300, and 400 °C. Among all 30P/BC-AC catalysts, the 30P/BC-AC(BA) provided the highest ethylene yield of 73.0–100.0% at the reaction temperature range of 250–400 °C, and the highest DEE yield of 45.2% at the reaction temperature of 200 °C. Therefore, the 30P/BC-AC(BA) catalyst is considered to be the most promising catalyst for ethanol dehydration for both ethylene production at 300–400 °C and DEE production at 200 °C.

### 2.4. Stability Test

**Figure 9:**
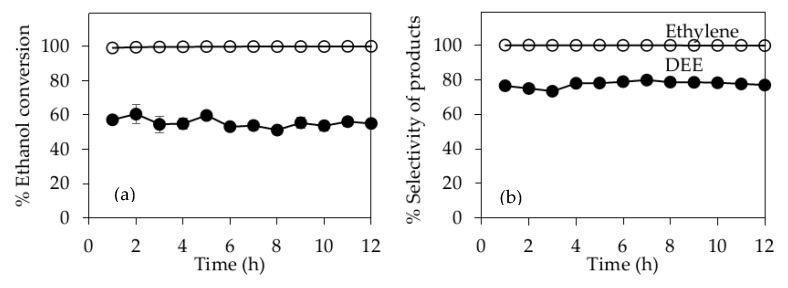
Stability test with a time-on-stream (TOS) for 30P/BC-AC(BA) catalyst at reaction temperature of 200 (●) and 400 °C (O): (**a**) Ethanol conversion, (**b**) Selectivity of products.

Because of its excellent catalytic performance, 30P/BC-AC(BA) was selected for the study of catalyst stability with a time-on-stream of 12 h. Figure 9 shows the ethanol conversions during the 12-h stability tests at reaction temperatures of 200 and 400 °C. At the low reaction temperature of 200 °C, the ethanol conversion and selectivity for DEE were quite stable at around 51.2–60.6% and 73.3–79.9%, respectively. At the high reaction temperature of 400 °C, the ethanol conversion was constant at 100%, with an ethylene selectivity of 100% during the 12-h stability test. These results indicate that the 30P/BC-AC(BA) catalyst has a very high catalytic activity and high stability in ethanol dehydration for the production of ethylene and DEE. In addition, 30P/BC-AC(BA) catalyst can be considered as a green catalyst because it is derived from bacterial cellulose and it is eco-friendly.

The performances of 30P/BC-AC(BA) and different catalysts using H_3_PO_4_ modification [31,32,33], different carbon-based catalysts [7,9,19,34], and other catalysts [35], with respect to ethanol conversion, ethylene selectivity, and DEE selectivity were compared as shown in Table 6. It has been demonstrated that 30P/BC-AC(BA) has excellent catalytic performance. 

## 3. Materials and Methods 

### 3.1. Materials

BC was synthesized by *A. xylinum* strain AGR 60. BC hydrogel was treated with 1% *w/v* NaOH for 24 h to remove bacterial cells, and then it was rinsed with deionized (DI) water until pH was 7.0 and was dried at 110 °C for 24 h.

### 3.2. Activated Carbon and Catalyst Preparation

BC-ACs were prepared by chemical activation with H_3_PO_4_ and/or KOH as activating agent using an incipient wetness technique. For one-step activation in the synthesis of BC-AC(A), dried BCs (size ≈1 × 1 × 0.1 cm^3^) were soaked with 85 wt % H_3_PO_4_ at 30 °C for 24 h, whereas, in the synthesis of BC-AC(B), dried BCs were soaked with 12 M of KOH at 30 °C for 24 h. After that, both impregnated BCs were dried at 110 °C for 24 h in a general electric oven (SNOL 67/350 LSP01, SNOL, Utena, Lithuania). The dried products were then carbonized at 500 °C in a muffle furnace (CWF 1100, Carbolite, Hope Valley, UK) (without any gas purging) for 1 h before cooling. For the two-step activation, the obtained BC-AC(A) and BC-AC(B) from the one-step activation were secondly chemical activated. For BC-AC(AB) synthesis, BC-AC(A) was secondly soaked with 12 M of KOH. For the BC-AC(BA) synthesis, BC-AC(B) was secondly soaked with 85 wt % of H_3_PO_4_. Then, they were dried and carbonized under similar conditions as previously described.

In order to be used as solid acid catalysts in ethanol dehydration reaction, the surface acidity of BC-AC(A), BC-AC(B), BC-AC(AB), and BC-AC(BA) was further modified by H_3_PO_4_ treatment. BC-ACs were impregnated with H_3_PO_4_ solution (30 wt % H_3_PO_4_) by the incipient wetness method. The solution of H_3_PO_4_ was dropped slowly onto 0.1 g of BC-ACs, and the BC-AC catalysts were then dehydrated in a general electric oven at 110 °C for 24 h.

### 3.3. Characterizations

The surface area, porosity and micro/meso pore size distribution of samples were determined by nitrogen (N_2_) physisorption-desorption using a micromeriticsm chemisorb 2750 pulse (Micromeritics, Norcross, GA, USA). The macropore volume and macropore size distributions were determined at pressure range of 0.1–61,000 psia by mercury porosimetry (AutoPore V 9600 version 1.03, Micrometrics, Norcross, GA, USA). The morphology of samples was determined by field emission scanning electron microscope (FESEM) (Model of JSM-7610F, JEOL, Oxford, MS, USA). The surface chemistry of samples was analyzed by the Fourier transform infrared (FTIR) spectroscopy (Nicolet 6700 FTIR spectrometer, Thermo Scientific, Waltham, MA, USA) and X-ray photoelectron spectrometer (XPS) (Axis Ultra DLD, Kratos Analytical, Manchester, UK). The acid properties of samples were investigated by the temperature-programmed desorption of ammonia (NH_3_-TPD) (Chemisorb2750 pulse, Micromeritics, Norcross, GA, USA). The methodologies used for the characterizations were previously reported in detail elsewhere [6,7].

### 3.4. Mass Transfer and Adsorption Capacity

The performances of mass transfer and adsorption of BC-ACs were evaluated for the removal of MB from aqueous solution at initial MB concentration of 600 mg/L. The initial pH of MB solution was adjusted to pH 7.0 by adding 0.1 M NaOH. BC-ACs (0.02 g) were added into the MB solution (40 mL) in a 100 mL Erlenmeyer flask. The adsorption experiment was conducted at 30 °C and 125 rpm using an incubator shaker (Innova 4330, New Brunswick Scientific, Edison, NJ, USA). For the kinetics and equilibrium study of MB adsorption, the solution of 0.1 mL of each flask was taken every 10 min for 1 h and every 30 min for 2 h. The solution samples were filtered passed through the filter paper. The concentrations of MB in the solutions were analyzed by a UV-visible spectrophotometer (UV-2450, Shimadzu, Japan) at a wavelength of 664 nm. A calibration curve was used to relate the optical density (OD) measurements to MB concentrations in the solutions. The amount of MB adsorbed per gram of adsorbent at any given time (q_t_, mg/g) was calculated by the following Equation (1):

(1) $$q_{t}=\frac{\left(C_{0}{\;-\;C}_{t}\right)V}{W}$$

where C_0_ and C_t_ (mg/L) are the initial concentration and the concentration of the MB solution at any time, t, respectively. V (L) is the volume of the solution, and (W) g is the weight of the adsorbent used.

### 3.5. Catalytic Ethanol Dehydration Reaction and Catalytic Stability of Catalysts

The ethanol dehydration reaction has been carried out under atmospheric pressure in a fixed-bed continuous flow micro-reactor using 0.1 g of catalyst at the temperature in the range of 200 to 400 °C. For catalytic stability test, the catalyst that provided the highest catalytic ethanol dehydration was selected to investigate its stability for ethanol dehydration reaction as a function of time on stream (TOS) at the reaction temperature of 200 °C and 400 °C for 12 h. The processes were previously reported in detail elsewhere [7]. The ethanol convsion, selectivity and yield were calculated by the following formulas:

(2) $$\mathrm{Ethanol}\;\mathrm{conversion}\;(\%)\;=\;\frac{\left(\mathrm{moles}\;\mathrm{of}\;\mathrm{ethanol}\;\mathrm{in}\;\mathrm{feed}\;-\;\mathrm{moles}\;\mathrm{of}\;\mathrm{ethanol}\;\mathrm{in}\;\mathrm{product}\right)}{\mathrm{moles}\;\mathrm{of}\;\mathrm{ethanol}\;\mathrm{in}\;\mathrm{feed}\;}\;\times \;100$$

(3) $$\mathrm{Selectivity}\;(\%)=\frac{\mathrm{moles}\;\mathrm{of}\;\mathrm{desired}\;\mathrm{product}\;\mathrm{formed}}{\mathrm{moles}\;\mathrm{of}\;\mathrm{total}\;\mathrm{product}\;\mathrm{formed}}\times 100$$

(4) $$\mathrm{Yield}\;(\%)=\frac{\mathrm{Ethanol}\;\mathrm{conversion}\times \mathrm{Selectivity}}{100}$$

## 4. Conclusions

BC was used as a carbon source for AC preparation due to its highly interconnected porous structure. A modified activation technique by two-step chemical activation was proposed. BC-AC(BA), which was prepared by the KOH activation, followed by the H_3_PO_4_ activation at a carbonization temperature of 500 °C showed the superior absorption performance. BC-AC(BA) has an interconnected multi-porous structure, composed of micropores (0.23 cm^3^/g), mesopores (0.26 cm^3^/g), and macropores (4.40 cm^3^/g) with porosity of 91.2% and BET surface area of 833 m^2^/g. According to the improved porous structure, the mass diffusion in the particle was enhanced, resulting in a higher absorption rate. The adsorption capacity was also increased to 593.4 mg of MB/g. The catalyst of 30P/BC-AC(BA) was shown to be an efficient catalyst for the dehydration of ethanol to ethylene and DEE. Ethylene selectivity of 82.6–100% and ethanol conversion of 88.4–100% were obtained by using 30P/BC-AC(BA) at a reaction temperature range of 250–400 °C, whereas DEE selectivity of 75.2% and ethanol conversion of 60.1% were obtained at the reaction temperature of 200 °C. Under a 12-h stability test, it was indicated that 30P/BC-AC(BA) was a very effective and selective catalyst with high stability in ethanol dehydration for the production of ethylene at 400 °C and the production of DEE at 200 °C.

## Figures and Tables

**Table 1:** The surface area, pore volume, and porosity of BC-ACs.

Activated Carbons	BET Surface Area ^a^ (m^2^/g)	Pore Volume (cm^3^/g)			Average Pore Size Diameter (nm)		Porosity ^b^ (%)
		^a^ Micro	^a^ Meso	^b^ Macro	^a^ Micro-Meso	^b^ Macro	
BC-AC(A)	1316	0.17	0.71	1.00	2.4	54	65.0
BC-AC(B)	340	0.13	0.09	3.30	2.6	1730	79.7
BC-AC(AB)	551	0.15	0.19	1.60	2.7	265	71.3
BC-AC(BA)	833	0.23	0.26	4.40	2.5	2,704	91.2

^a^ Determination by N_2_ physisorption. ^b^ Determination by mercury porosimetry.

**Table 2:** Acidity of BC-ACs and 30P/BC-ACs.

Samples	Acid Sites ^a^ (µmol NH_3_/g Catalyst)			
	Weak	Moderate to Strong	Total	Weak/Moderate to Strong
BC-AC(A)	206	109	282	1.9
BC-AC(B)	94	49	143	1.9
BC-AC(AB)	110	54	164	2.0
BC-AC(BA)	130	68	198	1.9
30P/BC-AC(A)	1141	294	1436	3.9
30P/BC-AC(B)	528	173	701	3.1
30P/BC-AC(AB)	609	209	818	2.9
30P/BC-AC(BA)	837	299	1136	2.8

^a^ Quantities of acid sites of catalysts were determined using temperature-programmed desorption of ammonia (NH_3_-TPD) with Fityk program calculation.

**Table 3:** Mass surface concentration determined by XPS quantitative analysis of BC-ACs and 30P/BC-ACs.

Samples	C (%)	O (%)	P (%)	N (%)	Si (%)
BC-AC(A)	74.3	20.7	3.1	1.9	-
BC-AC(B)	87.7	11.4	-	-	0.9
BC-AC(AB)	83.4	14.9	-	1.7	-
BC-AC(BA)	77.6	15.6	2.4	3.2	1.3
30P/BC-AC(A)	24.5	52.1	22.5	-	0.9
30P/BC-AC(B)	63.1	27.2	6.0	1.4	2.4
30P/BC-AC(AB)	55.9	30.5	11.5	-	2.2
30P/BC-AC(BA)	48.6	37.0	12.6	-	1.8

**Table 4:** Relative amount of phosphorus surface groups obtained from deconvolution of P 2p zone of XPS spectra of 30P/BC-ACs.

Samples	C–O–PO_3_ and (C–O)_3_PO (%)	C–PO_3_ and C_2_PO_2_ (%)
30P/BC-AC(A)	33.9	66.1
30P/BC-AC(B)	24.1	75.9
30P/BC-AC(AB)	30.3	69.7
30P/BC-AC(BA)	38.9	61.1

**Table 5:** Yield of ethylene, diethyl ether (DEE), and acetaldehyde (MeCHO) from ethanol dehydration at 200, 250, 300, and 400 °C.

Catalysts	200 °C			250 °C			300 °C			400 °C		
	Ethylene	DEE	MeCHO	Ethylene	DEE	MeCHO	Ethylene	DEE	MeCHO	Ethylene	DEE	MeCHO
30P/BC-AC(A)	7.1	34.5	2.5	24.9	32.1	4.7	90.5	6.8	1.1	100.0	0.0	0.0
30P/BC-AC(B)	0.7	16.4	1.0	19.0	34.5	8.6	85.9	2.2	2.8	94.8	0.3	0.6
30P/BC-AC(AB)	0.0	0.0	21.0	3.3	7.9	18.8	24.1	7.9	11.2	61.3	0.7	6.8
30P/BC-AC(BA)	9.0	45.2	1.5	73.0	13.5	1.9	95.8	2.6	0.8	100.0	0.0	0.5

**Table 6:** Comparison of catalysts for ethanol dehydration and their catalytic ability.

Catalysts	Reaction Temperature (°C)	Ethanol Conversion (%)	Ethylene Selectivity (%)	DEE Selectivity (%)	References
30P/BC-AC(BA)	200–400	60.1–100	15.0–100	75.2–0.0	This work
30P/BC-AC500	200–400	50.5–100	13.9–100	68.4–0.0	[7]
20HP-ZSM-5	250–400	25.0–99.0	3.0–99.0	96–0.0	[31]
5P/Al_2_O_3_	200–400	9.1–86.1	0.0–94.0	100–6.0	[32]
30PA-CeO_2_	400–500	16.0–59.7	96.1–99.2	0.2–0.1	[33]
25Al/BC-FD	200–400	41.3–65.7	1.1–43.3	40.0–43.3	[19]
HA2-800	200–350	2.0–100	34.0–100	65.0–0	[9]
AC_H420	400	39.1	69.4	29.9	[34]
SiO_2_	200–400	0.0–8.6	0.0–48.8	0.0–31.8	[35]
ZrO_2_	200–400	0.0–100.0	0.0–75.4	0.0–1.5	[35]
TiO_2_	200–400	0.6–100.0	0.0–29.7	83.8–0.0	[35]
